# Disruption of the mitochondrial network in a mouse model of Huntington's disease visualized by in-tissue multiscale 3D electron microscopy

**DOI:** 10.1186/s40478-024-01802-2

**Published:** 2024-06-05

**Authors:** Eva Martin-Solana, Laura Casado-Zueras, Teobaldo E. Torres, Gerardo F. Goya, Maria-Rosario Fernandez-Fernandez, Jose-Jesus Fernandez

**Affiliations:** 1 Department of Psychiatry, University of Pittsburgh, Pittsburgh, PA 15213 USA; 2 Advanced Microscopy Laboratory, University of Zaragoza, Zaragoza, Spain; 3 Instituto de Nanociencia y Materiales de Aragon (INMA), CSIC-Universidad de Zaragoza, 50018 Zaragoza, Spain; 4 Department of Condensed Matter Physics, University of Zaragoza, Zaragoza, Spain; 5 Department of Chemistry and Chemical Biology, Rutgers, The State University of New Jersey, Piscataway, NJ 08854 USA; 6 Spanish National Research Council (CSIC, CINN), Health Research Institute of Asturias (ISPA), 33011 Oviedo, Spain

**Keywords:** 3D electron microscopy, Volume electron microscopy, Electron tomography, Cryo-fixation, Huntington's disease, Mitochondria

## Abstract

Huntington’s disease (HD) is an inherited neurodegenerative disorder caused by an expanded CAG repeat in the coding sequence of huntingtin protein. Initially, it predominantly affects medium-sized spiny neurons (MSSNs) of the corpus striatum. No effective treatment is still available, thus urging the identification of potential therapeutic targets. While evidence of mitochondrial structural alterations in HD exists, previous studies mainly employed 2D approaches and were performed outside the strictly native brain context. In this study, we adopted a novel multiscale approach to conduct a comprehensive 3D in situ structural analysis of mitochondrial disturbances in a mouse model of HD. We investigated MSSNs within brain tissue under optimal structural conditions utilizing state-of-the-art 3D imaging technologies, specifically FIB/SEM for the complete imaging of neuronal somas and Electron Tomography for detailed morphological examination, and image processing-based quantitative analysis. Our findings suggest a disruption of the mitochondrial network towards fragmentation in HD. The network of interlaced, slim and long mitochondria observed in healthy conditions transforms into isolated, swollen and short entities, with internal cristae disorganization, cavities and abnormally large matrix granules.

**Supplementary Information:**

The online version contains supplementary material available at 10.1186/s40478-024-01802-2.

## Introduction

Huntington’s disease (HD) is an inherited neurodegenerative disorder caused by an expanded CAG repeat in the coding sequence of huntingtin protein (Htt). Symptoms typically emerge in middle age (35–45 years) and life expectancy post-onset is generally 15–20 years. Initially, the disease primarily affects the corpus striatum involving the selective neurodegeneration of medium-sized spiny neurons (MSSNs) [3]. Clinical manifestations include motor, cognitive, and psychiatric symptoms. Despite extensive research since the discovery of its genetic cause, the precise pathophysiological mechanisms of HD remain poorly understood [54]. Significantly, there is still no effective treatment for the disease [29, 42]. Consequently, identifying potential targets for therapeutic intervention in HD remains a top priority.

The expansion of a CAG repeat in the sequence of the gene coding for Htt leads to an abnormally expanded polyglutamine (polyQ) tract at the N-terminus of the protein [31]. The aberrant polyQ expansion induces conformational changes in Htt that increase its propensity to form aggregates, hallmarks of the pathology. Htt is a ubiquitous, mainly cytoplasmic, protein and it has been related to the endoplasmic reticulum (ER), mitochondria and Golgi complex [10]. Mutant Htt (mHtt), in either soluble or aggregate state, interferes with a wide spectrum of cellular functions, including transcription, cell traffic, autophagy and metabolism [3].

Mitochondria play a pivotal role in neurons as the organelles responsible for meeting the high energy demands necessary to support their physiological functions. Evidence of mitochondrial dysfunction has been identified in HD, with disturbances at the structural and functional levels [12, 41]. However, the precise causes and nature of this dysfunction remain unknown, even with conflicting results among different systems used for investigation [10, 25, 41, 43, 46, 52].

At the structural level, alterations in mitochondrial morphology and dynamics have been observed. Mitochondria are highly dynamic organelles that continuously undergo remodelling, fusion, fission and trafficking. There is evidence that mitochondrial dynamics is disrupted in HD, with an imbalance between fusion and fission that results in excessive mitochondrial fragmentation driven by increased GTPase activity of Drp1, a protein implicated in fission [7, 9, 12, 23, 40, 52, 56]. Ultimately, this may result in an abnormal distribution of mitochondria across the neuronal domains. Morphologically, cristae disorganization and swelling have been reported, which impair the mitochondrial capacity to produce energy [9, 25, 52, 56]. Additionally, recent findings describe the enlargement of mitochondrial matrix granules [64].

Electron microscopy (EM) stands as a classical technique for studying the cellular ultrastructure. Recent revolutionizing advances in three-dimensional (3D) imaging by EM and in sample preparation are enabling 3D ultrastructural studies of samples in their native context, preserved at close-to-physiological conditions and at a resolution of few nanometers. Electron Tomography (ET) and Focused Ion Beam Scanning Electron Microscopy (FIB/SEM) are two major 3DEM techniques that are allowing addressing fundamental questions in molecular and cell biology [8, 15, 32, 45]. ET relies upon a transmission electron microscope (TEM) and provides 3D ultrastructural information with resolution around 2–4 nm from biological samples with limited thickness (250–500 nm) [32, 45]. FIB/SEM overcomes this limitation by cyclically (i) milling a thin layer of the specimen using the FIB, followed by (ii) SEM imaging of the exposed surface. FIB/SEM can thus collect information from large 3D volumes (tens of microns thick) at a resolution around 5–10 nm [45, 65]. These 3DEM techniques can be combined through multiscale integrative approaches so as to enable comprehensive ultrastructural studies.

Sample preparation constitutes a crucial step in EM. Cryofixation, involving the rapid freezing of the sample (in milliseconds) and maintaining it hydrated in vitreous ice, ensures optimal structural preservation, avoiding artefacts induced by traditional chemical fixatives [26]. Cryofixation of thick samples (up to 200 microns thick) is accomplished by high-pressure freezing (HPF) to prevent the ice crystal formation [58, 59]. While observing pristine HPF samples under cryogenic conditions (cryo-ET, cryo-FIB/SEM) would be ideal, it remains challenging for tissues, although it is increasingly feasible for cell cultures. Cryo-ET would require thinning of the tissue sample by the technically demanding cryo-FIB lift-out technique and cryo-FIB/SEM volume imaging still suffers from significant charging artefacts and low contrast to identify specific cells within the tissue block. Consequently, the standard protocol for tissues to conduct comparative analysis among different conditions continues with freeze-substitution (FS) of the frozen water by an organic solvent and resin embedding [17]. The HPF/FS tissue sample can then be (i) cut into thin sections (up to 200–500 nm) for observation with ET or (ii) directly visualized in the FIB/SEM at room temperature.

3DEM combined with sample cryofixation is providing new insights into cellular compartments and their functions [5, 6, 18, 20, 32] and is gaining traction in the exploration of neurodegenerative diseases [30, 55, 67]. In HD in particular, these techniques are expanding our understanding of polyQ aggregates and subcellular alterations by working with in vitro samples or cultured cells [4, 19, 64, 66]. It is important to note, however, that these studies are conducted outside the strictly native context, as they do not deal with intact tissue samples.

In this study, we aim to explore the structural disturbances of mitochondria within their native tissue environment under the pathological conditions associated with Huntington's Disease. To this end, we conduct in situ 3D structural analysis of mouse brain tissue samples under optimal structural preservation conditions using a multiscale combination of advanced 3DEM techniques.

## Results

### FIB/SEM tomography reveals alterations in the mitochondrial network and morphology in a mouse model of HD

HD involves the selective degeneration of striatal MSSNs from the onset of the disease [3]. So, we aimed to analyze the organization and morphology of mitochondria in these neurons within their native brain tissue context to identify and characterize their disturbances in HD. To study such a complex and large structure as the mitochondrial network in a comprehensive manner and in 3D, we used FIB/SEM tomography. This imaging technology allows us to image large samples (in the range of tens or hundreds of microns) at a resolution of few nanometers.

Brain tissue samples from a 10-months old HD animal model (heterozygous zQ175) [37] and a corresponding littermate wild-type (WT) control were prepared with HPF/FS, as described in Materials and Methods. This preparation protocol ensures optimal structural preservation of tissue samples. The tissue blocks were examined by FIB/SEM tomography and stacks were acquired from cells compatible with morphological characteristics of striatal MSSNs [36]. A total of 8 and 5 MSSNs from the HD animal model and the control, respectively, were imaged. The acquired stacks represented volumes of a thickness in the range 5–30 microns.

**Fig. 1:**
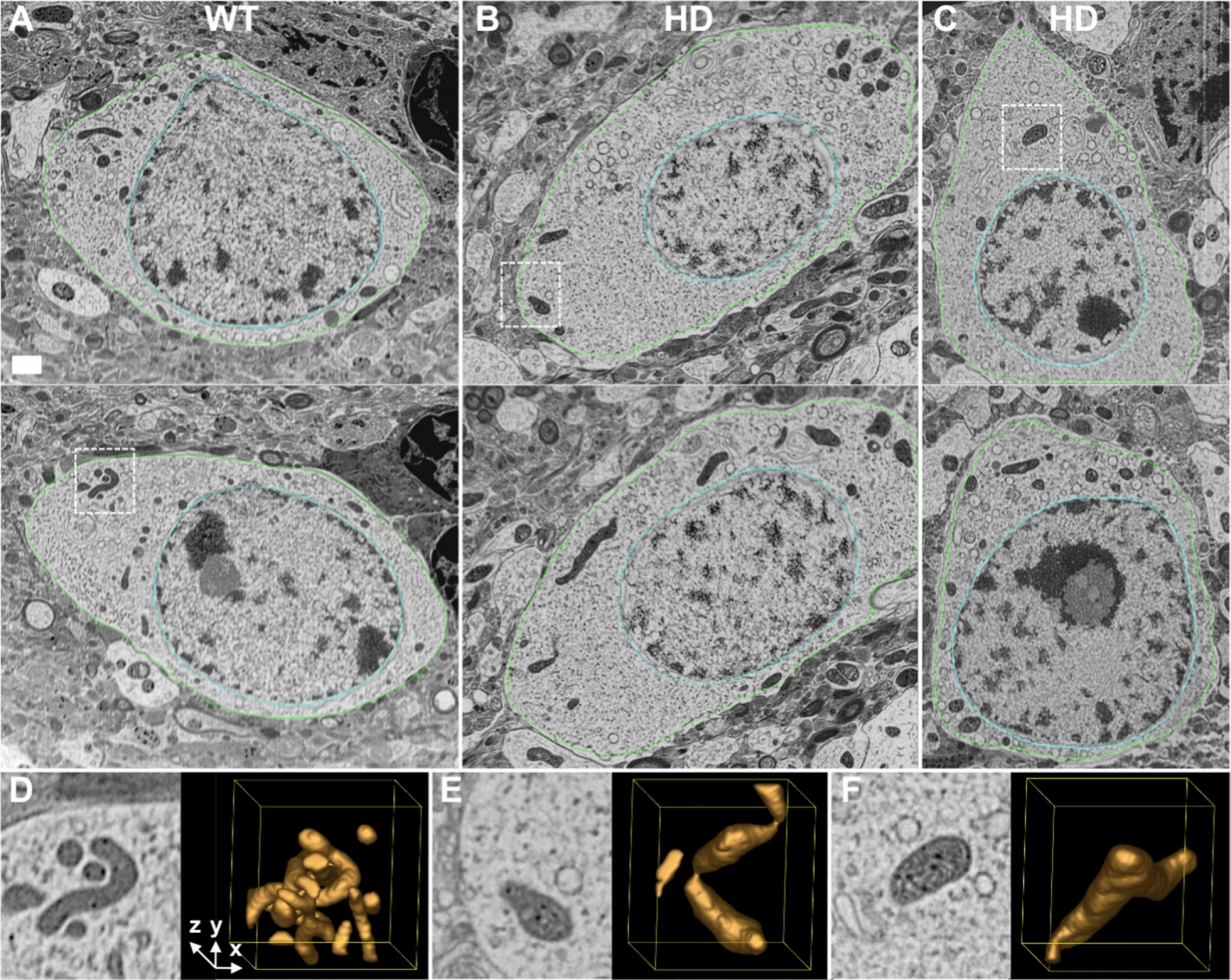
FIB/SEM tomography of MSSNs. **A**,**B**,**C** Two representative XY slices of one MSSN from the WT animal (**A**) and two MSSNs from the HD model (**B**,**C**) are shown. Green and cyan contours delineate the plasma and nuclear membranes, respectively. Mitochondria are identified as dark grey masses inside the lighter cytoplasm. Dashed boxes enclose selected cytoplasmic areas with representative mitochondria. Bar: 1 μm. **D**,**E**,**F** Magnified views of the dashed boxes in **A**,**B**,**C** respectively, are presented (left panels) along with 3D isosurface representations (right panels) of a volume of 2 × 2× 2 μm^3^ around those areas, thus showing the nearby context of those mitochondria

**Fig. 2:**
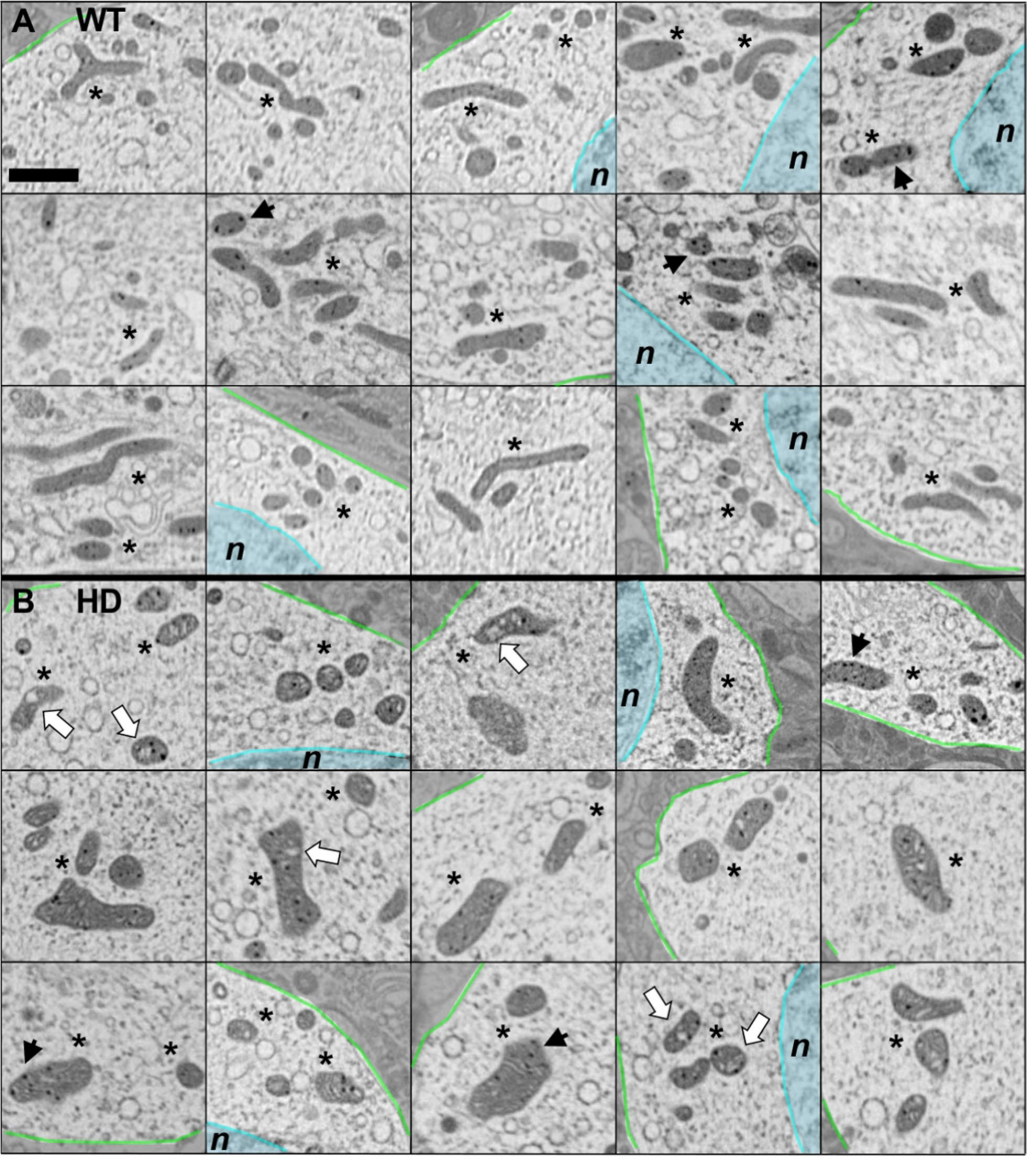
Mitochondria in slices of FIB/SEM volumes. Gallery of characteristic mitochondria (*) observed in 2D slices of all FIB/SEM volumes as dark grey masses within the lighter cytoplasm. **A** Mitochondria from 5 MSSNs from the WT animal. **B** Mitochondria from 8 MSSNs from the HD animal model. Mitochondrial granules are those black spots in the mitochondrial matrix (black arrowheads). To highlight the cytoplasm, nuclear areas (n) and extracellular space are shaded in cyan and grey colours, respectively, with the nuclear and plasma membranes delineated in cyan and green. Mitochondria in the HD model (**B**) present swollen and distorted shapes with separated cristae in comparison to the elliptical or long shapes in the WT animal (**A**). White arrows indicate some hollow areas with undefined content in the matrix of mitochondria in the HD model. Bar: 1 μm

Figure 1 presents representative 2D slices of three volumes, one MSSN from the WT animal (A) and two MSSNs from the HD model (B,C). In these slices, mitochondria are discernible as dark grey masses within the lighter spotty cytoplasm. Selected areas with representative mitochondria and their 3D context are also depicted in Fig. 1D,E,F, illustrating different scenarios such as interlaced mitochondria (D), potential fission process (E) or an isolated mitochondrion with abnormal shape (F). Figure 2 showcases a gallery of typical mitochondria collected from the slices of all the 13 volumes acquired. Figures 1 and 2 present consistent phenotypical mitochondrial features in the HD model and control. Mitochondria in the WT animal appear as relatively slim rods that, depending on the orientation in the 3D volume, may be observed as circular, elliptical or long shapes in the slices. Furthermore, they exhibit a nearly homogeneous inner density, except for the dark mitochondrial matrix granules, owing to their tightly packed cristae that are barely visible individually. In contrast, mitochondria in the HD model look as swollen, irregular and distorted shapes. Their inner density is not homogeneous and the cristae appear separated. Moreover, a distinctive feature is the presence of holes in the matrix, membrane-less open spaces among the cristae, often containing some indistinct material in their interior (Fig. 2, white arrows). Despite the resolution limitations, the matrix granules seem to be more noticeable than in the WT animal.

**Fig. 3:**
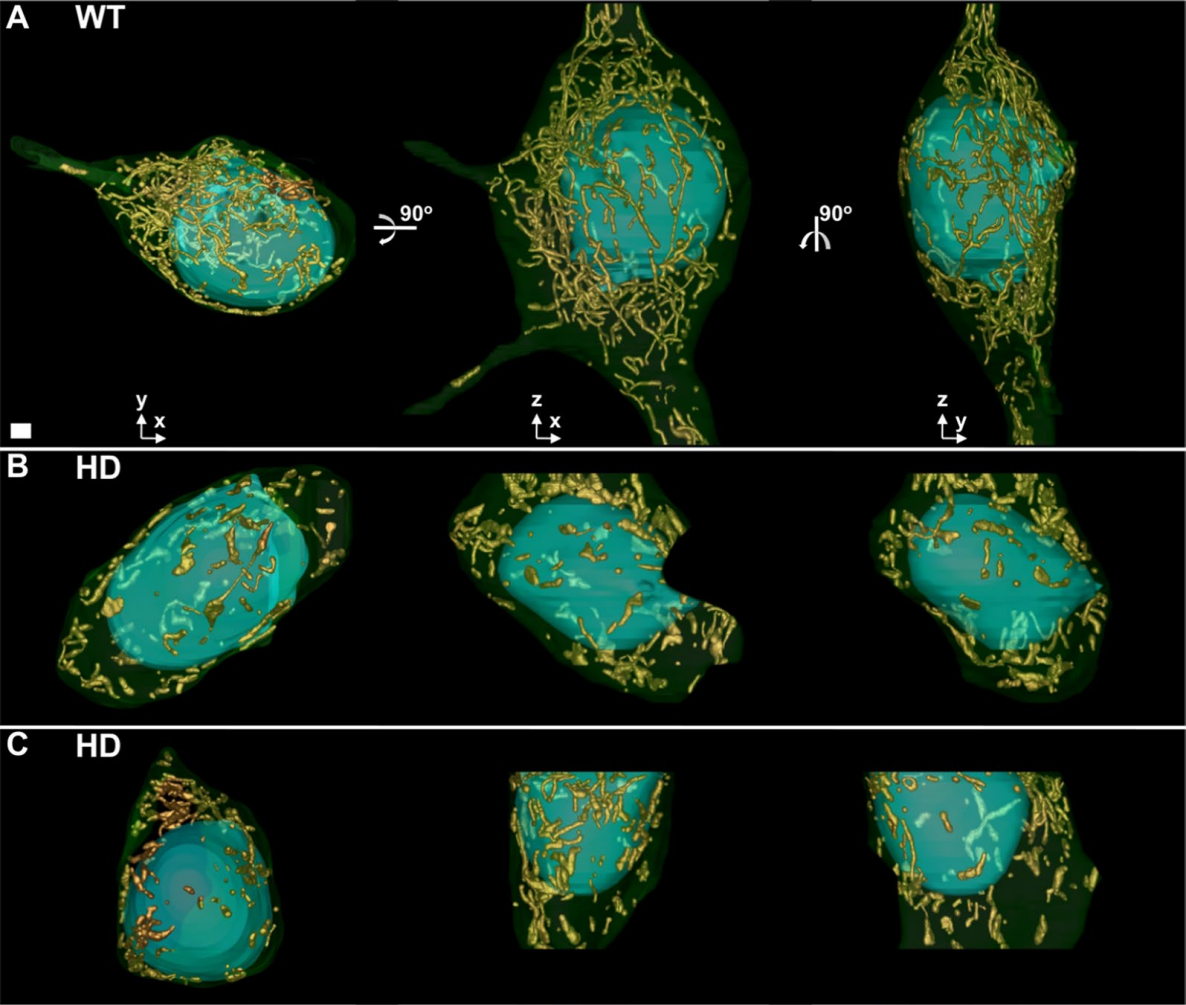
3D visualization of the FIB/SEM volumes. Three different views of the volumes from the MSSNs in Fig. 1**A**,**B**,**C** are presented in **A** (WT), **B** (HD) and **C** (HD), respectively. The leftmost views show the volumes with their Z axis running through the depth, a 90° rotation around the horizontal axis results in the views at the central panels, and a subsequent 90° rotation around the vertical axis produces the rightmost views. Segmented mitochondria are depicted with isosurface representation in gold colour. Plasma and nuclear membranes are displayed in 85% transparent green and 50% transparent cyan, respectively, allowing visualization of the mitochondria behind the nucleus. The missing wedge in the volume shown in **B** (central panel) is caused by a technical drift while FIB/SEM acquisition. Bar: 1 μm

Figure 3 presents 3D views of the three representative MSSNs shown in Fig. 1, featuring segmented mitochondria and delineated plasma and nuclear membranes (also see Supplementary Movies 1–3). The membranes are presented with transparency to ensure visibility of mitochondria at any side of the nucleus. This visualization of the entire cytoplasmic area that was imaged with the FIB/SEM microscope allows a more complete interpretation of the spatial distribution of mitochondria and their interrelationships. The thickness (size along the Z dimension) of those volumes was 25, 13 and 10 microns, respectively. In the WT animal, mitochondria appear as long and slim rods intricately interlaced and distributed throughout the cytoplasm, forming a complex mitochondrial network. In contrast, in the HD model, most mitochondria appear as isolated individual entities with irregular, rough and bumpy shapes and relatively short extensions, thus giving the impression of a disrupted mitochondrial network. While these alterations are somewhat recognizable in the 2D slices presented in Figs. 1 and 2, the full interpretation of these changes is only achievable through the 3D visualization of a significant area of the neuronal soma as in Fig. 3.

### Quantification of FIB/SEM data confirms the disruption of the mitochondrial network in a mouse model of HD

**Fig. 4:**
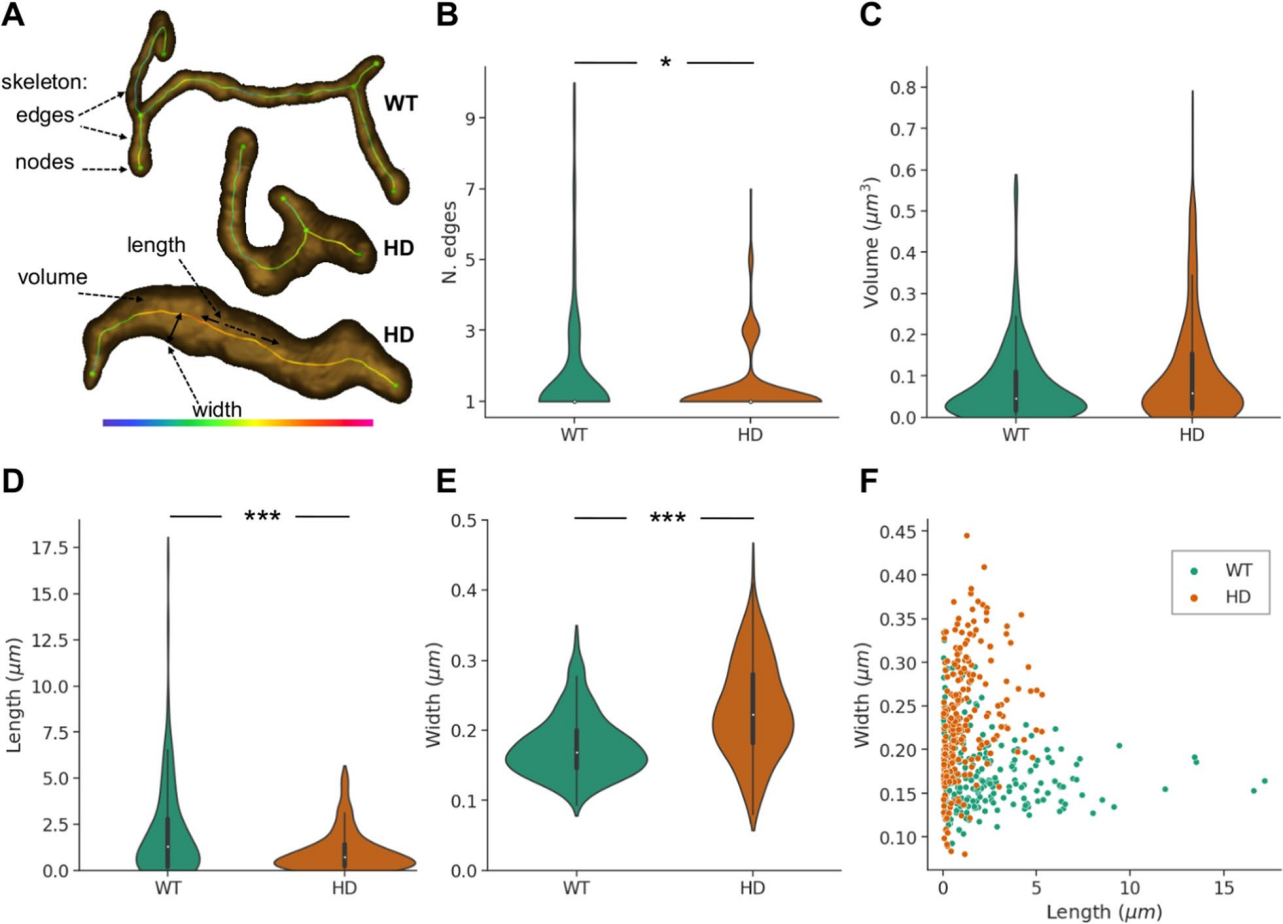
Quantification of mitochondrial alterations in FIB/SEM volumes. **A** Measurements. Each individual mitochondrion consists of its body and skeleton, where the skeleton comprises edges (mitochondrial segments or branches) and nodes (i.e. terminal ends and branching points). For each mitochondrion, the following measurements are obtained: number of edges, volume, length (sum of the length of its edges) and width (average of the local width -here shown with a colormap- along its edges). Illustrative examples of mitochondria from WT and HD animals are presented in semitransparent 3D isosurface representation with their skeleton overlaid. **B**–**F** Quantification plots. Comparison of measurements based upon 260 and 259 mitochondria from MSSNs of the WT animal and HD model. Violin plots show the distribution of the mitochondrial measurements: number of edges (**B**), volume (**C**), length (**D**) and width (**E**). A miniature boxplot is included inside the violin plots, with the box representing the interquartile range (between the first and third quartile), an additional quartile with the whiskers and the median with a white dot. The scatterplot (**F**) represents measurements (length and width) of all individual mitochondria. *p < 0.05; ***p < 0.0001

To conduct an objective quantification of the alterations, we devised a workflow that started with the automated segmentation of mitochondria in the volumes by means of an artificial-intelligence-based approach (see Materials and Methods, and Fig. 3 for the segmentation result). The binary volumes with the mitochondria segmented were then fed to MitoGraph software [24, 50, 61]. This program estimates the skeleton of each individual mitochondrion, given as edges (i.e. mitochondrial segments or branches) and nodes (i.e. terminal ends and branching points), and provides both length and local width of the individual edges (Fig. 4A). Finally, all the data from MitoGraph were compiled to provide measurements for each individual mitochondrion, namely number of edges (i.e. segments or branches), volume, length (sum of the length of its edges) and width (average of the width along the entire skeleton) (Fig. 4A).

To perform the quantification, we selected three representative FIB/SEM volumes, one MSSN from the control and two MSSNs from the HD model (Fig. 3), with a significant cytoplasmic volume of the neuronal soma, 796, 588 and 385 μm^3^, respectively. The automated segmentation procedure identified and segmented a total of 260, 140 and 119 mitochondria from the three volumes, respectively, which were then processed with MitoGraph. Subsequently, MitoGraph data were post-processed to obtain measurements for all individual mitochondria. For the statistical analysis, they were grouped into two categories: WT (260 mitochondria) and HD (259).

Figure 4 (B-F) presents the quantification results. While there are no significant differences in the total volume per mitochondrion between the phenotypes, there is a trend towards higher volume in HD (Fig. 4C). However, the other measurements (i.e. length, width and number of edges in Fig. 4D, 4E, 4B, respectively) indeed reveal statistically significant differences. Remarkably, mitochondria in the HD model are shorter (Fig. 4D) and exhibit a lower number of edges, mostly one (Fig. 4B), whereas in the WT counterpart they are notably longer and consist of a higher number of segments or branches, up to 10. This observation strongly supports the idea that an intricate network composed of mitochondria with many and relatively long branches is disrupted under pathological conditions in HD, transforming mitochondria into short, monolithic individual entities. This supports a scenario of mitochondrial fragmentation.

Moreover, Fig. 4E also shows that mitochondria under healthy conditions are relatively thin, mostly with a width around 0.17 μm. In contrast, in the HD model they are significantly thicker, with a broad width range (up to 0.44 μm) that accounts for irregular and bumpy shapes. Visualization of the length and width measurements of individual mitochondria in a scatterplot, as presented in Fig. 4F, clearly enables identification of the two phenotypes: short and thick in HD whereas long and slim in WT. These results explain why no significant differences in the total volume per mitochondrion are found, as described previously.

We also conducted the quantification by discriminating between the two MSSNs from the HD model. The results (Supplementary Figure S1) are consistent with the differences between the WT animal and the HD model described in the previous paragraphs.

In summary, these measurements and the results in Fig. 4 and in Supplementary Figure S1 faithfully reflect in objective and quantitative terms the alterations of the network and morphology observed in Figs. 1–3. They indicate disruptions in mitochondrial dynamics, with a tendency towards fragmentation, and disturbances in morphology, characterized by shorter and thicker (swollen) mitochondria under pathological conditions in HD.

### Electron tomography enables zoomed-in analysis of the mitochondrial disturbances and quantification of the altered matrix granules in HD

Although FIB/SEM tomography was valuable in visualizing the mitochondrial network and morphology, as described in previous sections, its resolution was still limited for studying fine details of the mitochondrial matrix, particularly granules. Therefore, we used ET as it enables identification and characterization of alterations in matrix granules thanks to the higher resolution power of this imaging technique.

For the ET experiments, we kept focused on striatal MSSNs from brain tissue samples and had available a 9-months old HD animal model (homozygous zQ175) and a corresponding WT control. The samples were prepared with HPF/FS, thereby ensuring optimal structural preservation as already described, and cut into ultrathin 250-nm-thick sections suitable for TEM and ET. A total of 120 sections from cells compatible with striatal MSSNs of the HD model and control were observed by TEM, showing consistent structural patterns in accordance with the observations by FIB/SEM already described. Subsequently, representative areas containing mitochondria from the HD model and the control were selected for examination through ET.

**Fig. 5:**
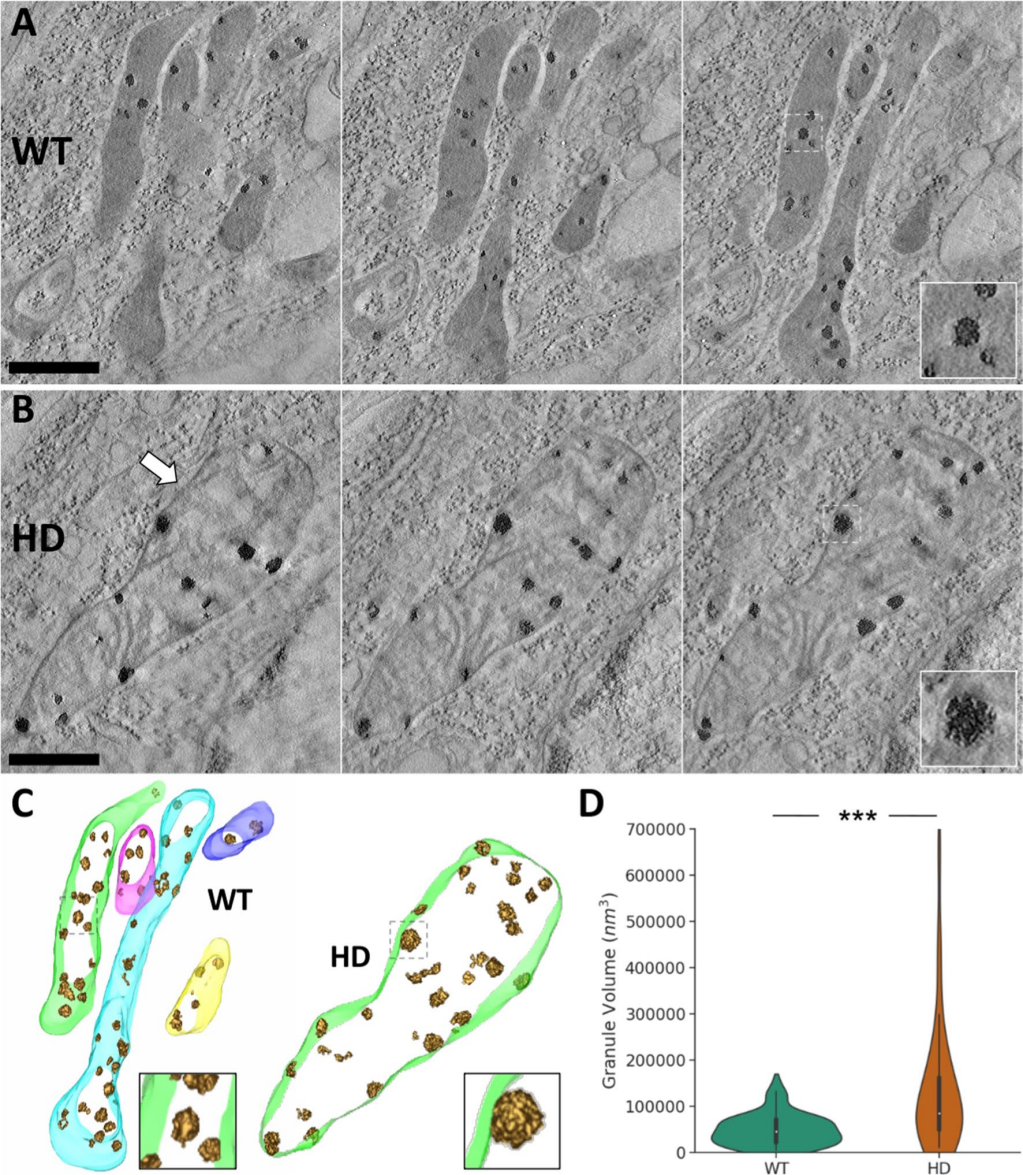
Electron tomography of mitochondria from MSSNs and analysis of matrix granules. **A**,**B** Tomograms of mitochondria from a WT animal (**A**) and a HD model (**B**). Three slices, separated by 22.12 nm, are shown for each tomogram. The arrow points to a hollow area with fuzzy content. Dashed boxes indicate granules magnified in the insets. Bar: 0.5 μm. **C** 3D visualization of the matrix granules within mitochondria. Granules are presented with isosurface representation in gold color and mitochondrial membranes are delineated in different semitransparent colors. **D** The distribution of the granule volumes obtained from 66 and 37 granules of the WT and HD animals, respectively, are presented with violin plots (right). Similar to Fig. 4, a miniature boxplot is included inside the violin plots and the median of the distributions denoted by a white dot. ***p < 0.0001

Figure 5 presents representative slices of the tomograms of the MSSNs from the WT animal (A) and the HD model (B) as well as their 3D visualization (C). The mitochondria in Fig. 5 exhibit features consistent with the previous FIB/SEM results shown in Figs. 1 and 2. In WT, a bundle of mitochondria was imaged, all of them being slim, rod-shaped and having relatively homogeneous density, except for the granules. The cristae are discernible, displaying a compact stacked organization. However, the mitochondrion from the HD model appears aberrantly swollen with a disrupted matrix where the cristae are abnormally disorganized and significant areas are devoid of material or have fuzzy content (Fig. 5, arrow).

ET allowed identification of matrix granules and thus a fair comparison between the phenotypes. Visually, the dense granules exhibit granulated textures, consisting of smaller structural units (Fig. 5, insets), whereas FIB/SEM only allowed visualization of matrix granules as black small spots (Fig. 2, black arrowheads). Furthermore, Fig. 5 already shows that the granules in the HD model generally appear larger than in the control. To carry out a quantitative analysis about their size, the individual granules were segmented in an objective manner (as described in Materials and Methods), yielding a total of 66 and 37 granules in the WT and HD model, respectively, and their volumes were then measured. Figure 5D presents the quantification results, confirming significantly enlarged granules in HD. In the WT animal the distribution of granule volumes is narrow and the median value (white dot in boxplots in Fig. 5D) is approximately 46,000 nm^3^ whereas in the HD model the distribution is much more dispersed and the median value is around 84,000 nm^3^. These two median volume values are equivalent to spheres with diameter 44 nm and 54 nm, respectively, which represents an enlargement factor around 1.23 × in the diameter of the mitochondrial granules of the HD model compared to WT. Finally, the number of matrix granules per nm^3^ of mitochondrial volume was measured from the segmented mitochondria and granules in Fig. 5, resulting in a decrease in the HD model (2.580 × 10^–7^ granules/nm^3^) with respect to the WT animal (8.097 × 10^–7^ granules/nm^3^).

## Discussion

There is evidence of mitochondrial dysfunction in HD, with implications at the functional and structural levels [12]. However, certain conclusions remain controversial, likely caused by the inherent differences between model systems or experimental approaches [41]. In this study, we employed a novel multiscale approach for a comprehensive 3D in situ structural study of the mitochondrial disturbances in a mouse model of HD. We analyzed MSSNs from brain tissue samples prepared with cryo-fixation-based methods to ensure structural preservation at close-to-native conditions. By combining various state-of-the-art 3D imaging technologies, we examined mitochondria over significantly large areas of the neuronal soma (FIB/SEM) and their inner details with sufficient resolution (ET). Finally, computational image processing facilitated quantification of the disturbances.

Our findings suggest a disruption of the mitochondrial network in HD, leading to fragmentation. This results in isolated, short, swollen and aberrantly shaped mitochondria dispersed throughout the cytoplasm, where the intricate network composed of interlaced, slim, long and branched mitochondria found in healthy conditions no longer exists. Moreover, upon closer examination, swollen mitochondria in HD exhibit disorganized cristae, internal hollow areas with fuzzy contents and abnormally large matrix granules. In contrast, in the control they appear dense with tightly stacked cristae and granules of moderate size.

One strength of our approach is that it considers large areas of the neuronal soma within intact tissue and works in 3D at a resolution of few nanometers, allowing for a more precise analysis of the mitochondrial network. Strategies based on 3D optical microscopy (e.g. confocal) enable analysis of the network within the entire cell volume [24, 50, 61], though at a limited resolution compared to electron microscopy. Working with 2D electron microscopy, hence from only partial views of mitochondria obtained from ultrathin sections of cells, is prone to misleading results concerning mitochondrial network, as it would be the case if conclusions on fragmentation were to be taken from Fig. 2. In our approach, image processing has also been important for quantification. However, to quantitatively reflect the visual results (Fig. 3), we had to employ elaborate metrics such as number of edges, length and width of mitochondria (Fig. 4). In this regard, the total volume per mitochondrion, which is the simplest measurement, proved to be a misleading metric that did not adequately reflect the evident alterations observed in Fig. 3. It showed no differences between control and HD (Fig. 4) simply due to the transformation of mitochondria from slim and long to swollen and short in HD. Regardless, our results of mitochondrial fragmentation are consistent with previous studies on other HD models or human samples, primarily based on 2D microscopy or molecular approaches [7, 9, 40, 56]. Conflicting results concerning fragmentation also exist [25], possibly attributable to differences in HD models, experimental strategies or the reasons just mentioned in this paragraph (i.e. working in 2D and/or with deceiving metrics).

Regarding morphology, the mitochondrial swelling and cristae disorganization observed in MSSNs (Figs. 2 and 5) align with all morphological reports thus far [9, 25, 38, 52]. Remarkably, we noticed hollow areas in the matrix with fuzzy content. They resemble mitochondrial vacuolization scenarios highlighted in previous works [25, 38]. They are also compatible with the mitochondrial pockets that emerge under perturbed mitochondrial dynamics to encompass the aberrant accumulation of mitochondrial RNA granules (MRG, fluid condensates that comprise essential components of the mitochondrial post-transcriptional pathway and mitoribosome biogenesis) [53].

The texture, number and volume of the matrix granules as well as the disturbances observed in intact brain tissue (Fig. 5) are consistent with recent findings in mouse neuronal cultures by cryo-ET [64]. These granules, identified as calcium phosphate deposits, are related to the role of mitochondria in subcellular calcium homeostasis. They absorb the excess of cytoplasmic calcium and store it in the form of granules [44, 47], whose larger size needs accommodation in the matrix through remodelling cristae. An excessive calcium influx can trigger mitochondrial swelling, depolarization and eventual collapse [47, 57], potentially contributing to the observed aberrant morphology. The composition of these calcium phosphate granules has been determined by elemental analysis [63] and studies on mitochondrial calcium uptake capacity with cryo-fixed samples [28, 57]. Therefore, the abnormal size of the granules in the HD model may be linked to the excess of basal intracellular calcium [11, 49, 51] or the controversial lower calcium uptake capacity [44, 46] associated to this disease. It is important to note the fact that visualization and quantification of these granules are only feasible with cryo-fixed samples [28, 57, 63, 64], as chemical fixation methods result in their extraction [48].

Recently, new proteomics data have revealed an enrichment of proteins involved in RNA processing in iPSC-derived cultured neurons from HD patients, suggesting that the matrix granules are MRGs [64]. Therefore, it is possible that both, calcium phosphate and components of MRGs, coexist as constituents of the observed electron-dense granules. Alternatively, the abnormally accumulated MRGs might be located within the mitochondrial pockets [53] often observed in our FIB/SEM volumes, as described above.

In conclusion, our approach combining tissue cryofixation, multiscale 3D electron microscopy and image processing has enabled the direct visualization, holistic analysis and quantification of the mitochondrial disruptions in HD within the native brain context. Our results support the evidences of mitochondrial fragmentation in HD research derived from partial or 2D approaches and might serve to reconcile some of the conflicting views. Our innovative approach opens new avenues for the in situ analysis of the disturbances of subcellular compartments and the identification of therapeutic targets in HD and in other neurodegenerative diseases.

## Materials and methods

### Animals

A stable colony of the zQ175 mice [37] was established through founders donated by the Cure Huntington’s Disease Initiative (CHDI) and sourced from Jackson Laboratory Inc. The zQ175 line is a knock-in model bred on a C57BL/6 J background featuring an endogenous murine *HTT* gene with a chimeric human/mouse exon 1 containing approximately 190 CAG repeats (B6.12951-Htt < tm1Mfc < 190JChdi). Heterozygous and homozygous mice and wild-type (WT) control counterparts were bred to maintain a stable colony within the animal facility of the Centro Nacional de Biotecnologia (CSIC). They were provided with food and water ad libitum. All experiments complied with Spanish and European legislation and were in accordance with the ethical guidelines established by the Spanish National Research Council (CSIC) ethics committee concerning animal experimentation.

### Sample preparation based on HPF/FS

Brain tissue samples were prepared for ET and FIB/SEM imaging following our established protocols designed to ensure optimal structural preservation. These procedures are primarily based on high-pressure freezing and freeze-substitution (HPF/FS), as previously described [17]. In short, mouse brains were dissected immediately post-mortem and 200-μm-thick sagittal slices were cut using a tissue slicer (Stoelting, Co.). Striatal samples were promptly extracted, placed onto a flat specimen carrier, and then subjected to high-pressure freezing within a Leica EMPACT2 device. The samples were further processed with freeze-substitution of frozen water to methanol, including 0.5% uranyl acetate, and were subsequently embedded in Lowicryl resin HM20 with a Leica AFS2 EM FSP system.

For visualization in the TEM and for ET, Sects. (250 nm thick) were obtained from the resin-embedded samples using a Leica Ultracut EM-UC6 ultramicrotome, and placed on Quantifoil S7/2 grids.

### FIB/SEM imaging

FIB/SEM imaging was done on a FEI/ThermoFisher Scientific Helios NanoLab Dual-Beam 650 at the LMA node of the Spanish ICTS ELECMI. Regions of interest were identified by visual inspection of the sample surface with the SEM. Areas with cells compatible with striatal MSSNs were selected based on morphological criteria [36] and FIB/SEM stacks were then acquired. Prior to imaging, the areas were protected with a carbon deposit (approximately 1 μm thick) performed by FE/FIBID (Focused electron and ion beam-induced deposition). The milling was performed using a slice thickness of 15 to 25 nm. Image acquisition was done at 2 kV and current 1.6 nA using a TLD detector in BSE mode. Stacks of hundreds of images representing a sample thickness of 5 to 30 microns were acquired with a pixel size at the specimen level in the range of 8 to 11 nm.

### TEM and ET imaging

A conventional JEOL JEM-1011 transmission electron microscope (100 kV) was used to screen the 250-nm-thick sections, check the integrity of the tissue samples, and select areas of interest. Cells compatible with striatal MSSNs were selected based on morphological criteria [36] for subsequent ET and analysis. The magnification was set to 10 K and 30 K for identification of neurons and mitochondria, respectively.

Tomographic data were acquired by taking series of images from the sections while tilting them within a range of ± 60° at 1° interval around a single tilt-axis. The tilt-series were acquired using a Thermo Fisher Scientific/FEI Tecnai G2 (200 kV) equipped with a CCD camera. The pixel size at the specimen level was 0.79 nm. For processing, visualization and analysis, the images were rescaled with a binning factor of 4. Prior to ET, grids were incubated in a solution of 10-nm diameter colloidal gold (EM.BSA 10, Electron Microscopy Sciences, Hatfield, PA, USA) to facilitate subsequent image alignment.

### Image processing of FIB/SEM stacks

Acquired stacks were first processed with contrast enhancement and noise reduction [16, 21]. The resulting stacks were then subjected to alignment with IMOD [27]. For 3D visualization and analysis, the stacks were rescaled to isotropic voxel size [22]. Delineation of plasma and nuclear membranes was done manually with IMOD tools to define the cytoplasmic area of the neuron. Semantic segmentation of mitochondria in the cytoplasm was performed with automated deep-learning procedures. To this end, a 2.5D U-net neural network was implemented to operate on 2D slices plus the immediate neighbour slices to predict the location of mitochondria using Dragonfly software (Comet Technologies Canada Inc.) [33]. The U-net had a depth level of 5 and worked on patches of 64 × 64. The training was volume-specific and was conducted in two steps. For the first step, training data was objectively produced by a computational procedure consisting of edge-preserving filtering of the FIB/SEM stacks with anisotropic non-linear diffusion [14, 39] followed by thresholding on density and on size of connected components [34, 35]. This procedure enabled preliminary segmentation of mitochondria. The U-net was then trained using a subset of 100 slices of these stacks, with 2 × data augmentation, using a batch size of 32 and a maximum number of epochs of 25 with early stopping criterion on a 20% slice subset that acted as a validation subset. After that first training step, the labels predicted for the training subset were manually revised to produce a new training data. The U-net network was then subjected to a second training step using the new data, continuing from the previous state of the network and using the same training hyperparameters. The final trained network was then applied to the whole stack to derive, after some manual revision, the definite segmented mitochondria.

Quantitative analysis of the segmented mitochondria was then carried out with MitoGraph [24, 50, 61] and in-house programs. The binary, segmented tomograms were processed with MitoGraph to decompose the individual mitochondria into their body and their skeleton comprising edges (mitochondrial segments or branches) and nodes (either mitochondrial ends or branching points) and to obtain measurements of the length of edges and the local width (distance from the edge points to the mitochondrial surface). An in-house program was developed to process these data and to provide measurements for individual mitochondria (number of edges, volume, length as the sum of the length of their edges, and width as the average of the width along the entire skeleton) for statistical analysis and for visualization with IMOD.

### Image processing of ET data

Alignment of the tilt-series and 3D reconstruction of the tomograms were conducted using IMOD software [27] and Tomo3D [1, 2] applying standard protocols [13]. Alignment was based on the colloidal gold beads as fiducial markers using IMOD. Tomographic reconstruction relied on weighted back-projection (WBP) using a filter that simulates an iterative reconstruction method (SIRT).

Delineation of mitochondrial outer membranes in the tomograms was done manually with IMOD tools. Automated semantic segmentation of mitochondrial matrix granules was done with edge-preserving filtering of the tomograms with anisotropic non-linear diffusion [14, 39] followed by density thresholding. This procedure was enough to segment the granules owing to their significantly different density in comparison with the rest of the mitochondrial matrix.

### Statistical analysis and plotting

Statistical analyses were performed with Python using the Pingouin package [60]. The comparisons were carried out based on the Mann–Whitney test as the Shapiro–Wilk tests indicated that all data were non-normally distributed. Plots were generated with the Seaborn package [62].

### Supplementary Information

Supplementary material 1

Supplementary material 2

Supplementary material 3

Supplementary material 4

## Data Availability

The data of this study are available from the corresponding authors on reasonable request.
