# Supplementary material for: Disruption of the mitochondrial network in a mouse model of Huntington's disease visualized by in-tissue multiscale 3D electron microscopy
Source: Acta Neuropathol Commun. 2024 Jun 5;12:88. doi: 10.1186/s40478-024-01802-2 (PMC11151585; doi:10.1186/s40478-024-01802-2)
Supplement: Supplementary file 1 — Supplementary material 1 [file 40478_2024_1802_MOESM1_ESM.pdf]

Supplementary Information

Disruption of the mitochondrial network in a mouse model of Huntington's Disease visualized by in-tissue multiscale 3D electron microscopy

E Martin-Solana, L Casado-Zueras, TE Torres, GF Goya, MR Fernandez-Fernandez, JJ Fernandez

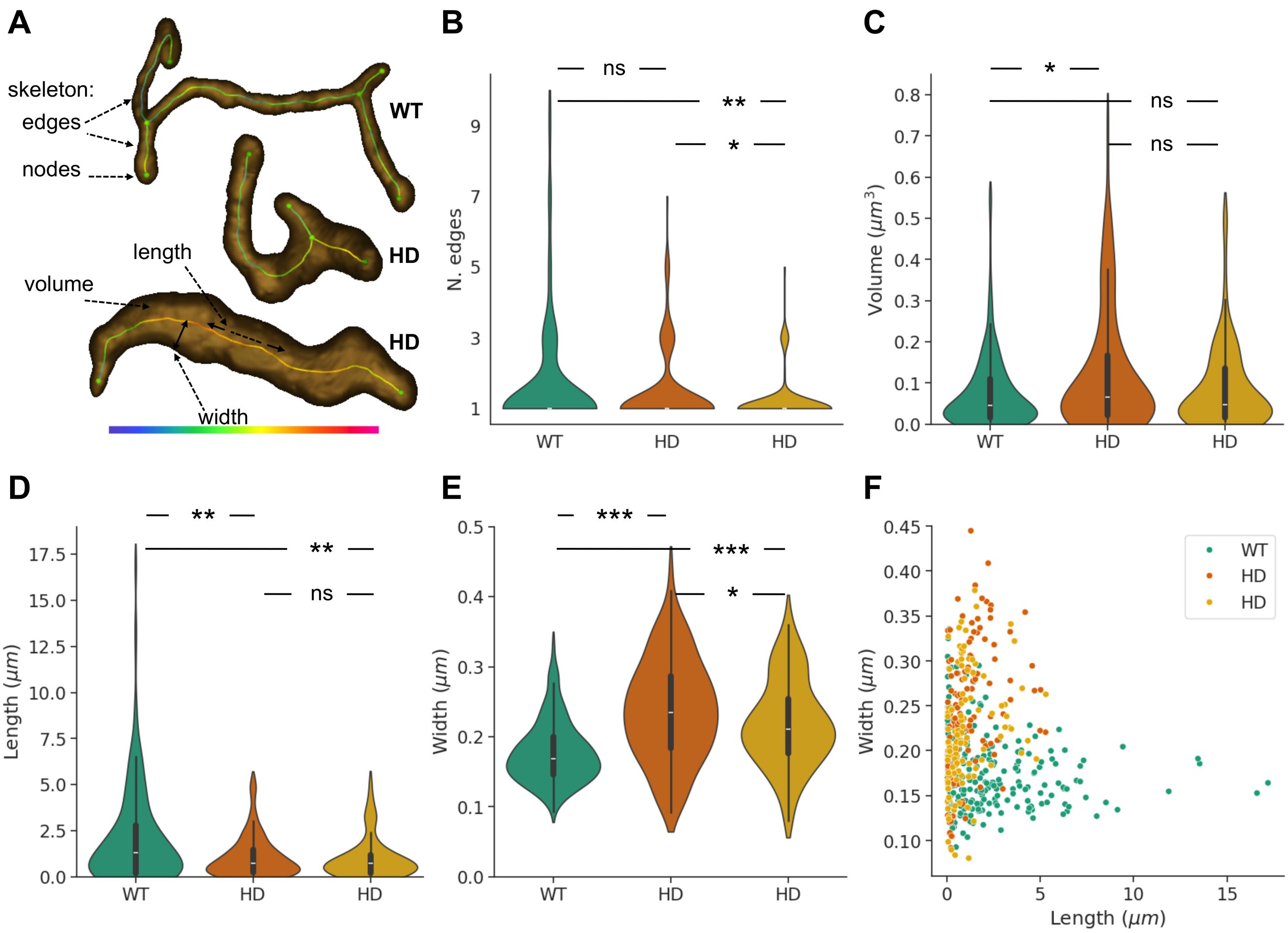

Supplementary Figure S1. Quantification of mitochondrial alterations in FIB/SEM volumes by separating the three neurons (1 WT, 2 HD). (A) Measurements. Each individual mitochondrion consists of its body and skeleton, where the skeleton comprises edges (mitochondrial segments or branches) and nodes (i.e. terminal ends and branching points). For each mitochondrion, the following measurements are obtained: number of edges, volume, length (sum of the length of its edges) and width (average of the local width -here shown with a colormap- along its edges). Illustrative examples of mitochondria from WT and HD animals are presented in semitransparent 3D isosurface representation with their skeleton overlaid. **(B-F) Quantification plots.** Comparison of measurements based upon 260, 140 and 119 mitochondria from the MSSN of the WT animal (Figure 3A) (plotted in green) and the two MSSNs from the HD model, Figure 3B (plotted in orange) and Figure 3C (plotted in yellow), respectively. Violin plots show the distribution of the mitochondrial measurements: number of edges (B), volume (C), length (D) and width (E). A miniature boxplot is included inside the violin plots, with the box representing the interquartile range (between the first and third quartile), an additional quartile with the whiskers and the median with a white dot. The scatterplot (F) represents measurements (length and width) of all individual mitochondria. ns: not significant ($p>0.05$); *$p<0.05$; **$p<0.01$; ***$p<0.0001$.
